# Territorial CT Perfusion Burden and Outcome After Aneurysmal Subarachnoid Haemorrhage: A Retrospective Single-Centre Cohort Study

**DOI:** 10.3390/diagnostics16182979

**Published:** 2026-09-15

**Authors:** Felix Schlicht, Bogdana Tokareva, Helge C. Kniep, Lukas Meyer, Matthias Bechstein, Gregor Peter, Christian Heitkamp, Laurens Winkelmeier, Vincent Geest, Alexander Heitkamp, Safouh Muzaiek, Luca Meucci, Maxim Bester, Fabian Flottmann, Caspar Brekenfeld, Hanno S. Meyer, Lasse Dührsen, Jens Fiehler, Christian Thaler

**Affiliations:** 1 Department of Diagnostic and Interventional Neuroradiology, University Medical Centre Hamburg-Eppendorf (UKE), 20246 Hamburg, Germanyg.peter@uke.de (G.P.); c.heitkamp@uke.de (C.H.);; 2 Department of Neurosurgery, University Medical Centre Hamburg-Eppendorf (UKE), 20246 Hamburg, Germany

**Keywords:** aneurysmal subarachnoid haemorrhage, delayed cerebral ischaemia, cerebral vasospasm, computed tomography perfusion, functional outcome, risk stratification

## Abstract

**Background**: Delayed cerebral ischaemia remains a major cause of secondary brain injury after aneurysmal subarachnoid haemorrhage (aSAH). Although computed tomography perfusion (CTP) is widely used for vasospasm surveillance, perfusion abnormalities are commonly reported only as present or absent, potentially overlooking the prognostic relevance of their extent. We investigated whether a territory-based perfusion burden score is associated with outcome more closely than binary perfusion deficit assessment. **Methods**: This retrospective single-centre cohort study included 179 patients with aSAH who underwent at least one CTP examination during hospitalization. CTP was performed according to clinical indication rather than at predefined time points; 448 examinations were evaluated. Two blinded neuroradiologists assigned one point per affected vascular territory (score range 0–12), and the maximum score during hospitalization was analysed. Outcomes were functional outcome at 6 months (modified Rankin Scale 0–2 versus 3–6), new cerebral infarction at discharge, and in-hospital mortality. Multivariable logistic regression models were built by forward selection from prespecified covariates (age, Hunt and Hess grade, modified Fisher grade, intracerebral haemorrhage, external ventricular drainage, and aneurysm treatment modality). **Results**: Territorial perfusion burden was independently associated with poor functional outcome (adjusted odds ratio 1.17 per point, 95% confidence interval 1.02–1.33) and new cerebral infarction (adjusted odds ratio 1.36 per point, 95% confidence interval 1.19–1.55). Any perfusion deficit was associated with new infarction (odds ratio 8.88, 95% confidence interval 3.31–23.87) but not with functional outcome. **Conclusions**: In this selected cohort under CTP surveillance, the extent of perfusion impairment was associated with functional outcome and infarction, whereas binary deficit assessment was associated with infarction only. These preliminary findings suggest that territorial perfusion burden may refine risk stratification, but prospective validation with standardized imaging time points is required before the score can be used as a prognostic tool.

## 1. Introduction

Delayed cerebral ischaemia (DCI) is the main preventable secondary injury following aneurysmal subarachnoid haemorrhage (aSAH) [1]. Despite intensive monitoring and advances in treatment, reliable prediction of DCI remains difficult [2]. Besides neurological examinations, current surveillance strategies rely on transcranial Doppler (TCD) and vascular imaging with computed tomography angiography (CTA) or digital subtraction angiography (DSA), as recommended in international guidelines [3,4]. These diagnostic tools visualize the degree of arterial narrowing, which is associated with DCI, but they do not provide direct insight into perfusion status or microcirculatory changes in the brain tissue [5,6].

The pathophysiological mechanisms behind DCI are multifactorial. Besides arterial narrowing, factors such as collateral status, microcirculatory dysfunction, microthrombosis, neuroinflammation, and cortical spreading depolarizations may play a role in the development of DCI [7,8]. This may partly explain why there is only a partial correlation between angiographic vasospasm and downstream tissue perfusion, and why some patients develop DCI without severe narrowing, whereas other patients do not despite vasospasm. Evidence from endovascular stroke treatment indicates that vasospasm can be substantially reduced by pharmacological measures without a corresponding improvement in functional outcome, underscoring that vasospasm alone is an imperfect surrogate for clinically relevant cerebral ischaemia [9]. This has led to growing interest in the direct assessment of parenchymal perfusion as a potentially more informative approach for risk stratification, particularly in comatose patients or when conventional diagnostic tools are not conclusive [5,6].

CT perfusion (CTP) imaging enables quantification of parameters such as cerebral blood flow (CBF), cerebral blood volume (CBV), and mean transit time (MTT). Multiple studies have shown that perfusion abnormalities after aSAH are strongly associated with the development of DCI and with worse functional outcome, providing a higher diagnostic value than vessel imaging alone [5,10,11]. However, the clinical use of CT perfusion is limited by a lack of standardized perfusion thresholds, different post-processing methods, and simplified clinical reports that only state the presence or absence of a perfusion deficit, all of which lower its prognostic value. Therefore, a more structured and reproducible assessment strategy is needed.

In this study, we applied a territory-based perfusion score in a cohort of patients with aSAH who underwent CTP during vasospasm surveillance. We hypothesized that the extent of perfusion impairment is associated with infarction and functional outcome more closely than the conventional dichotomous perfusion assessment. Because clinical DCI could not be assessed systematically in this retrospective cohort, new cerebral infarction, i.e., the radiological component of the consensus definition of DCI [1], and functional outcome served as endpoints.

## 2. Materials and Methods

### 2.1. Study Design and Patient Population

We performed a retrospective, single-centre cohort analysis including patients with aSAH admitted to our department between January 2019 and June 2024. The diagnosis of aSAH was confirmed either by CT imaging or lumbar puncture, and the aneurysmal source was identified by CTA or DSA. Inclusion required at least one CTP examination during hospitalization and available follow-up imaging. A total of 179 patients met these criteria. Because CTP was performed according to clinical indication (Section 2.2), patients with an uncomplicated course in whom no indication for CTP arose were not included, and the cohort represents a selected population under CTP surveillance rather than an unselected aSAH population. The Ethics Committee of the Ärztekammer Hamburg, Germany (reference number 2022-300245-WF), reviewed the study protocol and determined that formal ethical approval was not required for this retrospective study, which was conducted in accordance with the Declaration of Helsinki [12]. The requirement for informed consent was waived due to the retrospective nature of the study. All data were de-identified and anonymized.

Baseline demographic data, vascular risk factors, and aneurysm characteristics were collected from electronic medical records. The severity of aSAH was classified according to the Hunt and Hess and modified Fisher scales. Aneurysm size and localization were recorded from the initial angiographic studies and categorized as anterior or posterior circulation. Information on clinical presentation at admission, time of symptom onset, treatment procedures (microsurgical clipping or endovascular treatment), the need for external ventricular drainage (EVD), intra-arterial spasmolysis, and length of hospital stay was retrieved from electronic medical records.

### 2.2. Imaging Protocol

All patients underwent serial multimodal CT examinations following institutional monitoring standards. CTP was not performed at predefined time points but according to clinical indication, so that the number and timing of examinations varied between patients and reflected the individual clinical course. In total, 448 CTA/CTP studies were evaluated, corresponding to a median of two per patient (IQR 1–3). Main indications included new or progressive neurological deficits or decreased consciousness (47%), increased transcranial Doppler (TCD) velocities (26%), defined as a mean velocity > 140 cm/s or a doubling of the measurements within 24 h, and vasospasm screening in sedated or comatose patients who could not be assessed clinically (27%).

The first CTP was typically performed after a median of 4 days (IQR 2–8) post-onset, coinciding with the period of highest risk for DCI, which usually peaks between days 4 and 10 after aSAH.

CTP data were acquired using a 128-slice multidetector CT scanner (SOMATOM Force; Siemens Healthineers, Forchheim, Germany) with 70 kV, 150 mAs, and 50 mL of iodinated contrast (Imeron 400; Bracco Imaging Deutschland GmbH, Konstanz, Germany) injected at 6 mL/s.

### 2.3. Perfusion Analysis and Scoring

CT perfusion data were processed using the CT Neuro Perfusion module on a syngo.via workstation (software version VB80F; Siemens Healthineers, Forchheim, Germany). All 448 CTP examinations were independently analysed by two experienced readers (with 5 and 11 years of neuroradiological experience) blinded to all clinical parameters and outcomes; discrepancies were resolved by consensus, and the consensus rating was used for all analyses. Interrater agreement for the detection of a perfusion deficit per examination was assessed using Cohen’s kappa statistic with 95% confidence intervals; agreement for the numerical score was not assessed. Focal perfusion abnormalities due to the primary haemorrhagic event or to surgical intervention, as identified on the images of the CTP dataset, were not scored.

**Figure 1:**
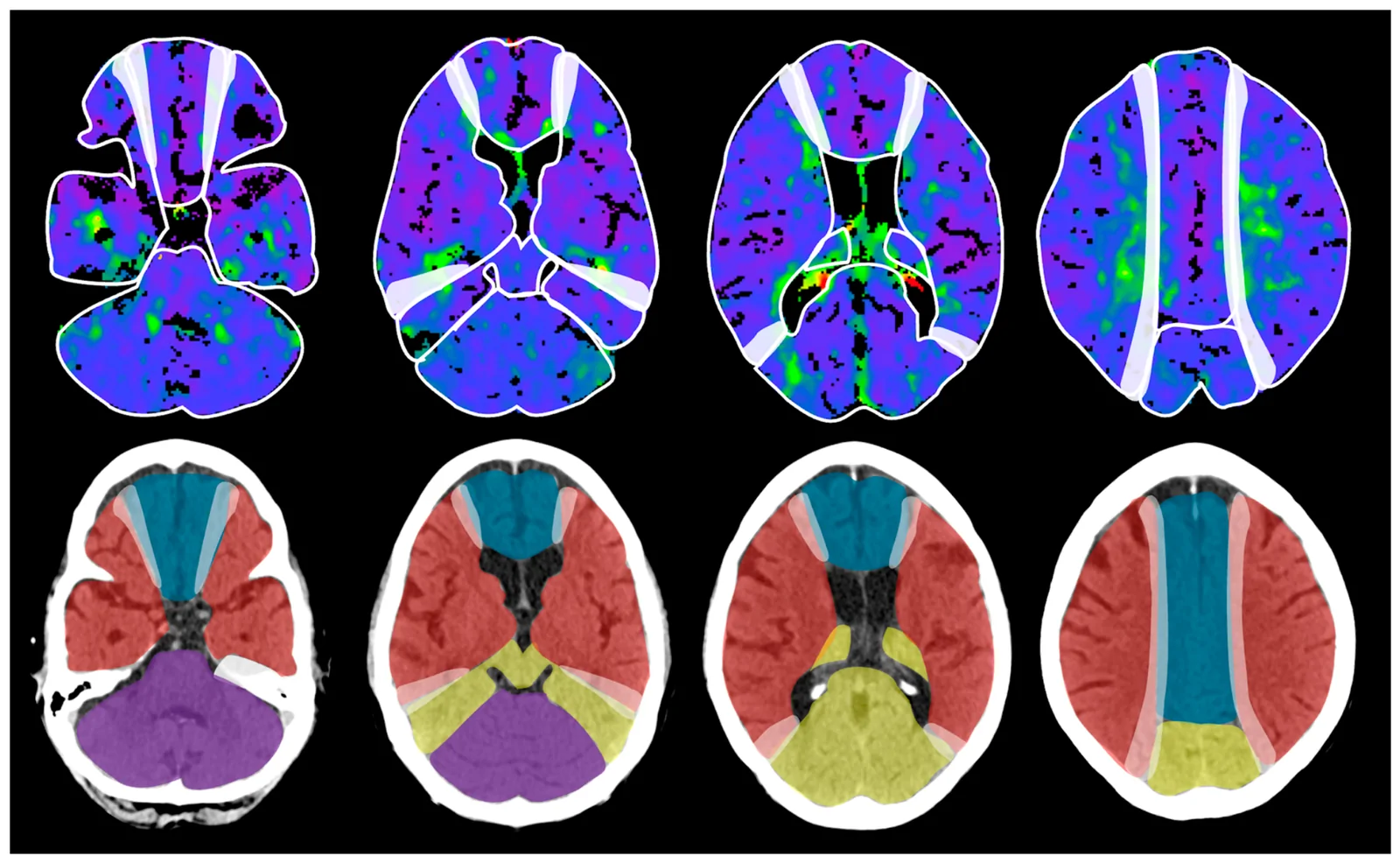
Vascular territory definition used for the territorial computed tomography perfusion burden score. Representative time-to-drain perfusion maps at four axial levels (posterior fossa and temporal lobes, midbrain and tentorium, basal ganglia and lateral ventricles, centrum semiovale) illustrate the standardized vascular territory assignment applied in the scoring system. Anterior cerebral artery territories (blue), middle cerebral artery territories (red), posterior cerebral artery territories (yellow), and cerebellar territories (purple) are delineated. Included border zones are limited to the anterior (anterior-middle cerebral artery) and posterior (middle-posterior cerebral artery) cortical watershed regions and are shown as semi-transparent overlays; deep border zones were scored but not included in the analyses. Territorial boundaries follow published arterial territory atlases [13,14].

Perfusion abnormalities were allocated to 12 predefined territories: the anterior, middle, and posterior cerebral artery territories of each hemisphere, the two cerebellar hemispheres, and the anterior (between the anterior and middle cerebral artery territories) and posterior (between the middle and posterior cerebral artery territories) cortical border zones of each hemisphere. Territorial boundaries followed published arterial territory atlases [13,14] and were applied at four axial levels (Figure 1). Deep (internal) border zones were also scored but were not included in the TPBS or in the analyses. The border zones were scored as separate territories because watershed hypoperfusion is a typical and often early manifestation of haemodynamic compromise in vasospasm [15,16] and would otherwise either be disregarded or be counted as an entire arterial territory. Each territory contributed one point irrespective of its size.

Regions with delayed perfusion were identified on Tmax and time-to-drain (TTD) maps, both of which have shown favourable diagnostic performance for vasospasm-related hypoperfusion [11,17]. Delayed perfusion was defined as a visually apparent regional prolongation on Tmax and TTD maps compared with the contralateral hemisphere and with perfusion patterns typically observed in patients without vasospasm. A territory was rated as affected only if the prolongation was present on both maps, followed a territorial or border-zone distribution rather than a patchy pattern, and was reproducible over adjacent slices. Abnormalities attributable to artefacts (motion, beam hardening from coils, clips, or ventricular catheters, venous contamination, poor bolus arrival), to physiological asymmetry, or to pre-existing parenchymal lesions were not scored. In bilateral or symmetric abnormalities, the unaffected territories of the same examination, typically the posterior circulation and the cerebellum, served as internal reference. No absolute perfusion thresholds were applied.

**Figure 2:**
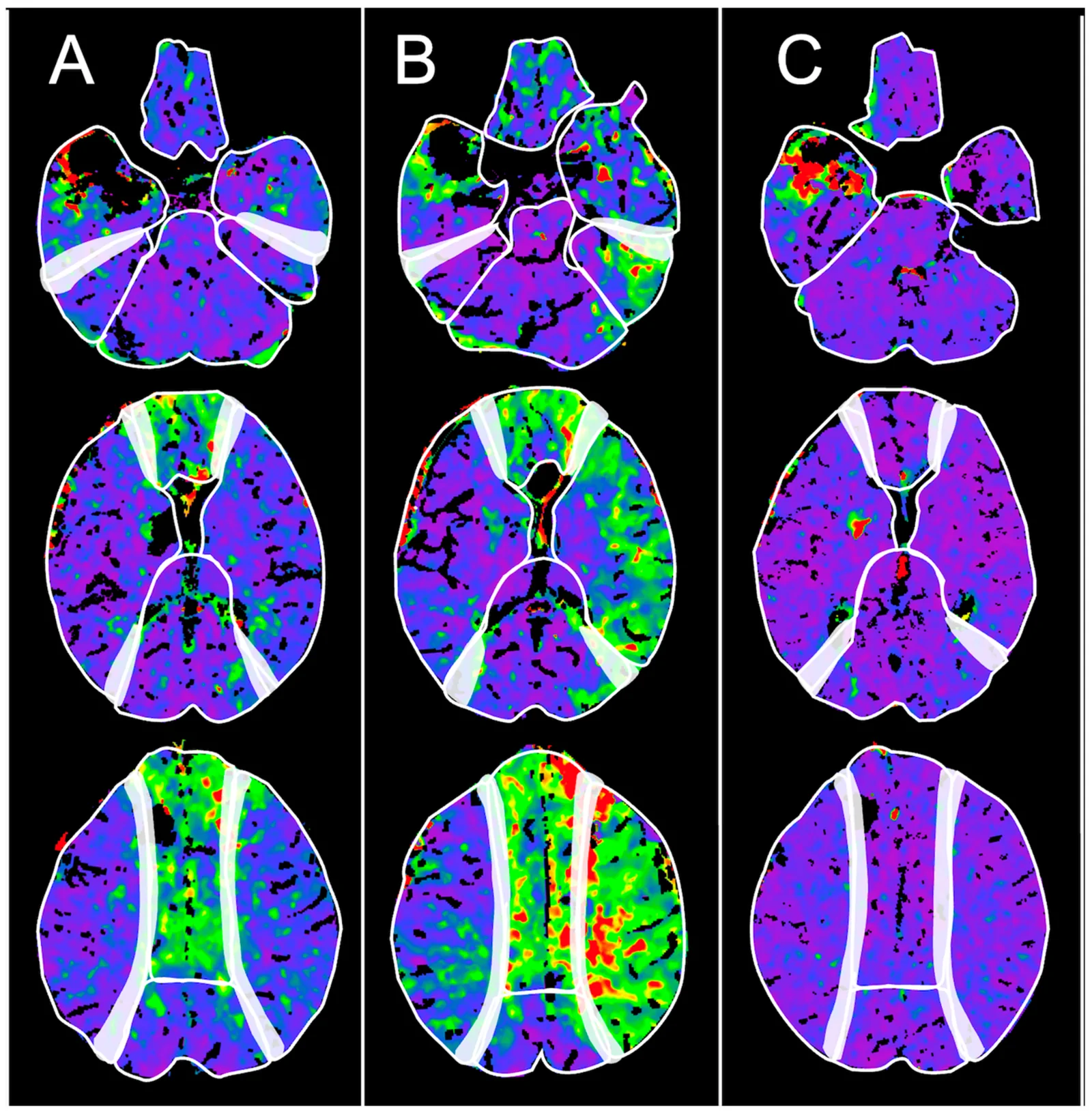
Temporal evolution of territorial perfusion abnormalities in a patient with vasospasm after aneurysmal subarachnoid haemorrhage. Serial time-to-drain perfusion maps obtained at three time points (**A**–**C**) are displayed at three axial levels per time point. Evaluated vascular territories are outlined in white, and included border zones are indicated by semi-transparent overlays. (**A**) Bilateral anterior circulation perfusion delay is present on day 5, corresponding to a territorial perfusion burden score of 2. (**B**) The perfusion abnormality progresses on day 9 to involve the bilateral anterior and the left middle cerebral artery territories with additional anterior and posterior border-zone impairment, corresponding to a territorial perfusion burden score of 5. (**C**) The perfusion abnormalities resolve completely on day 11, corresponding to a territorial perfusion burden score of 0. The maximum score of the serial examinations (5, panel (**B**)) was used as the patient-level score.

For each examination, the territorial perfusion burden score (TPBS; range 0–12) was calculated as the number of affected territories, and the maximum TPBS of all examinations during hospitalization was used as the patient-level score (Figure 2). The maximum was chosen because it represents the peak of haemodynamic compromise, which is the information that determined clinical management. Because the number and timing of examinations were indication driven, neither a fixed-time-point score nor a time-weighted burden could be derived consistently, and a baseline score would have been similarly unsuitable, since the first examination was obtained at variable intervals after onset. For comparison with the conventional dichotomous assessment, a binary variable (any perfusion deficit versus no deficit) was derived from the same data.

### 2.4. Clinical Outcome Assessment

To detect new ischaemic lesions that were not related to the initial aneurysm treatment, CT at admission, after endovascular or neurosurgical aneurysm treatment, and at the time of patient discharge was reviewed by two neuroradiologists (consensus rating) blinded to the patient characteristics and outcomes. New cerebral infarction was defined as a new hypodensity on CT or diffusion restriction on MR imaging at discharge that was not present after aneurysm treatment and not attributable to aneurysm treatment, ventricular catheter placement, or intracerebral haemorrhage, corresponding to the radiological component of the consensus definition of DCI [1]. Clinical DCI according to the consensus criteria was not assessed systematically, because a substantial proportion of patients were sedated or comatose during the vasospasm period. Functional outcome was assessed using the modified Rankin Scale (mRS) after 6 months and dichotomized as good (mRS 0–2) or poor (mRS 3–6); a 6-month assessment was available in all 172 patients included in the outcome analyses. The mRS was obtained at the outpatient follow-up visit or, when a visit was not possible, by structured telephone interview or from the medical records.

### 2.5. Statistical Analysis

Descriptive statistics were used to summarize baseline characteristics and imaging findings. Continuous variables are presented as mean ± standard deviation (SD) or median with interquartile range (IQR), and categorical data as absolute and relative frequencies. Normal distribution was evaluated visually. Group comparisons were performed with the unpaired *t*-test for continuous data, the χ^2^ test for categorical data, and the median test for ordinal variables.

Binary logistic regression was used to analyse the association of the perfusion findings with poor outcome (mRS 3–6) and new infarction at discharge. Candidate covariates were prespecified on the basis of clinical relevance and prior literature (age, Hunt and Hess grade, modified Fisher grade, intracerebral haemorrhage at admission, treatment modality, and the need for an EVD) and entered as continuous or categorical variables as appropriate. Multivariable models were built by forward conditional selection from this prespecified set (entry *p* < 0.05, removal *p* > 0.10); covariates that were not retained are reported as such in the tables. The perfusion findings were modelled either as a binary variable (any perfusion deficit) or as the TPBS (continuous, per one-point increase) in separate models to avoid collinearity; entering the TPBS as a continuous variable assumes a constant change in the log-odds per additional territory, and linearity was not tested formally. Intra-arterial spasmolysis was not entered as a covariate, because it was initiated in response to the perfusion findings and is therefore a consequence of the exposure rather than a confounder; adjusting for it would remove part of the association under study rather than confounding [18,19].

**Figure 3:**
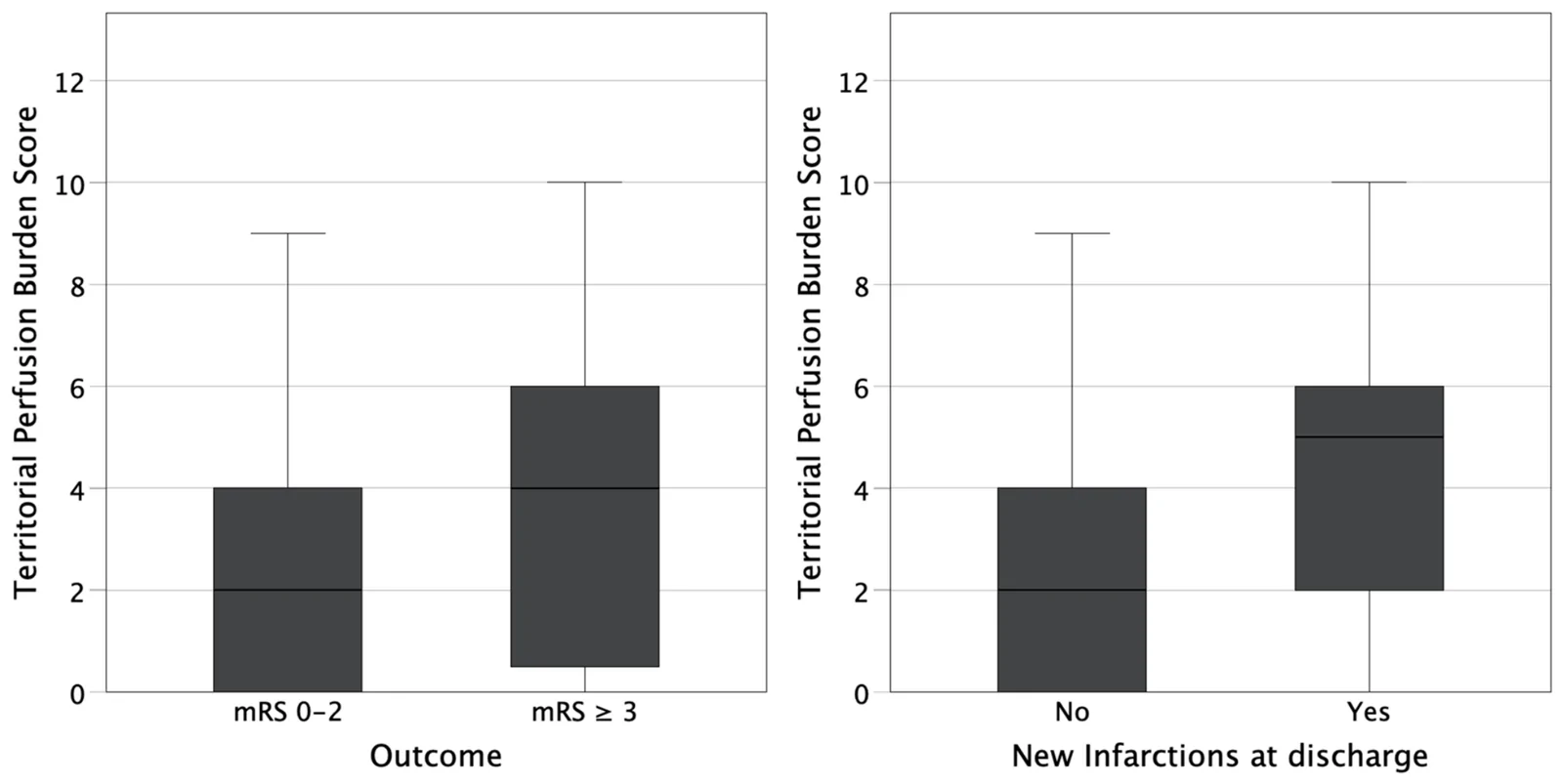
Association of territorial perfusion burden with functional outcome and infarction. Maximum territorial perfusion burden score during hospitalization stratified by functional outcome at follow-up (modified Rankin Scale [mRS] 0–2 vs. mRS ≥ 3, **left**) and by new cerebral infarction at discharge (no vs. yes, **right**). A higher territorial perfusion burden score was associated with poor functional outcome and with the occurrence of new infarction. *p*-values reflect univariable between-group comparisons.

Results are expressed as odds ratios (OR) with 95% confidence intervals (CI). Seven patients without outcome data were excluded from the outcome analyses (*n* = 172), whereas all 179 patients contributed to the analysis of in-hospital mortality. A formal comparison of model discrimination between the binary and the territorial perfusion models was not performed, because the study was designed as an exploratory association study and not as a prognostic model development study. All statistical analyses were performed using IBM SPSS Statistics, version 30 (IBM Corp., Armonk, NY, USA). A two-sided *p*-value < 0.05 was considered statistically significant. Figure overlays and annotations were prepared using Microsoft PowerPoint, version 2508 (Microsoft 365; Microsoft Corp., Redmond, WA, USA). Figure 3 was generated using Python, version 3.12 (Python Software Foundation, Wilmington, DE, USA) with the Matplotlib library (open-source; matplotlib.org).

### 2.6. Use of Generative Artificial Intelligence

ChatGPT (version GPT-5; OpenAI, San Francisco, CA, USA) was used to assist with language editing, spelling correction, and adjustment of word count in the manuscript text after all analyses had been completed. The tool was not involved in the study design, data analysis, or interpretation of results. All AI-assisted edits were reviewed and verified by the authors, who take full responsibility for the content of the manuscript.

## 3. Results

### 3.1. Study Population

We included 179 patients with aSAH who fulfilled the inclusion criteria. The mean age was 57.5 years (SD 13.0), and 68 patients (38%) were female. In total, 448 CTA/CTP examinations were available, with a median of 2 studies per patient (IQR 1–3). The first imaging was performed at a median of 4 days after aSAH onset (IQR 2–8). Imaging indications included new neurological deficits or decreased consciousness in 210 studies (47%), elevated transcranial Doppler velocities in 117 (26%), and routine surveillance in comatose patients in 121 (27%) studies.

Hypertension was present in 84 patients (47%), diabetes mellitus in 7 (4%), and 66 (37%) were active smokers. The median Hunt and Hess score was 3 (IQR 2–4), and the modified Fisher score was 3 (IQR 2–4). Baseline characteristics stratified by the presence of perfusion deficits are summarized in Table 1.

### 3.2. Perfusion Findings

Perfusion deficits were detected in 122 of 179 patients (68%). The examination yielding the maximum TPBS was obtained at a median of 8 days (IQR 4–11) after aSAH. The interrater agreement for the detection of a perfusion deficit per examination was excellent (κ = 0.93; 95% CI 0.90–0.97).

Hunt and Hess and modified Fisher scores did not differ significantly between patients with and without perfusion deficits (*p* = 0.68 and *p* = 0.84, respectively). Aneurysm location was associated with perfusion abnormalities, with perfusion deficits being more frequent in patients with internal carotid artery aneurysms (with vs. without perfusion deficits: 13.9% vs. 3.5%; *p* = 0.04) and less frequent in patients with middle cerebral artery aneurysms (10.7% vs. 26.3%; *p* = 0.01).

Patients with perfusion deficits were less frequently treated by surgical clipping and more frequently by endovascular therapy compared with patients without perfusion deficits (20.5% vs. 45.6% and 79.5% vs. 54.4%, respectively; both *p* < 0.001). Intra-arterial spasmolysis was performed substantially more often in patients with perfusion deficits (73.8% vs. 28.1%; *p* < 0.001). Other baseline variables, including age, sex, and vascular risk factors, did not differ between patients with and without perfusion deficits (Table 1).

### 3.3. Association with Infarction and Outcome

The results of the univariable and multivariable regression analyses are summarized in Table 2 (functional outcome) and Table 3 (new cerebral infarction). New cerebral infarctions not associated with surgical or endovascular treatment occurred in 88 of 172 evaluable patients (51.2%). In the univariable regression analysis, the binary classification of a perfusion deficit (OR 8.88, 95% CI 3.31–23.87) as well as the TPBS (OR 1.36 per point, 95% CI 1.19–1.55) were associated with new cerebral infarctions at discharge. Two multivariable models were built: the first including the binary classification of a perfusion deficit and the second including the TPBS. Both variables remained significantly associated with new infarction in the separate multivariable models. None of the other candidate variables, such as age, Hunt and Hess score, modified Fisher score, ICH at admission, treatment modality (clipping vs. endovascular therapy), or necessity of an EVD, was significantly associated with new cerebral infarctions at discharge, and none was retained in the multivariable models (Table 3).

Favourable functional outcome (mRS 0–2) was achieved in 79 of 172 evaluable patients (45.9%), and in-hospital mortality was 12.8% (23 of 179 patients). In the univariable analysis, higher age (OR 1.05, 95% CI 1.02–1.07), higher Hunt and Hess score (OR 1.98, 95% CI 1.53–2.57), higher modified Fisher score (OR 2.04, 95% CI 1.48–2.81), ICH at admission (OR 2.84, 95% CI 1.48–5.45), necessity of an EVD (OR 5.14, 95% CI 2.12–12.50), and higher TPBS (OR 1.18 per point, 95% CI 1.05–1.32) were associated with poor outcome, whereas the binary classification of a perfusion deficit was not (OR 1.70, 95% CI 0.88–3.29).

Two multivariable models were built by forward selection from the candidate variables. In the TPBS model, TPBS (aOR 1.17 per point, 95% CI 1.02–1.33), age (aOR 1.05, 95% CI 1.02–1.08), and Hunt and Hess grade (aOR 1.90, 95% CI 1.45–2.48) remained independently associated with poor functional outcome, whereas modified Fisher grade, ICH at admission, treatment modality, and EVD were not retained. In the deficit model, age, Hunt and Hess grade, and modified Fisher grade were retained, whereas the binary perfusion deficit was not. In-hospital mortality did not differ between patients with and without perfusion deficits (13.9% vs. 10.5%; *p* = 0.64). No multivariable model was built for mortality because of the small number of events (*n* = 23). A detailed overview of the univariable and multivariable regression analyses is provided in Table 2 and Table 3 and illustrated in Figure 3.

## 4. Discussion

In this retrospective study of patients with aSAH under CTP surveillance, we evaluated a territory-based perfusion burden score to quantify the extent of perfusion abnormalities. The score was independently associated with new infarction and with functional outcome, whereas the conventional dichotomous assessment was associated with infarction but not with functional outcome. In the adjusted models, each additional affected territory was associated with a 36 percent increase in the odds of infarction and a 17 percent increase in the odds of poor functional outcome. These findings suggest that the extent of perfusion impairment carries prognostic information that is not captured by the presence of a deficit alone, and that perfusion abnormalities after aSAH exist along a spectrum of severity rather than as a binary finding.

Perfusion abnormalities after aSAH are an established marker of delayed cerebral ischaemia and poor outcome [5,10,20]. Perfusion imaging is therefore recommended as part of multimodal surveillance in current guidelines [3,4]. However, there is no consensus on when perfusion abnormalities should indicate treatment escalation to prevent the occurrence of DCI. Quantitative thresholds for parameters such as CBF or MTT have been proposed, but absolute values vary substantially across scanners and post-processing platforms, which limits their clinical application [6,21,22]. Unlike threshold-based perfusion approaches, the TPBS is less dependent on absolute perfusion values, which may reduce software-related variability. Qualitative visual assessments can identify ischaemic regions [23] and are associated with delayed infarction more closely than angiographic vasospasm [20], yet a binary classification does not reflect the extent of the deficit and therefore lacks prognostic differentiation [24].

The territorial perfusion burden score is intended to address these shortcomings. By grouping perfusion abnormalities into predefined vascular territories, it captures the regional and multifocal pattern typical of DCI pathophysiology, which involves microcirculatory dysfunction, impaired autoregulation, inflammation, and cortical spreading depolarizations as described by Dreier et al. [7,8]. Unlike threshold-based methods [17,25,26] or qualitative binary assessments of perfusion deficits [25], this approach quantifies the regional extent of perfusion impairment in a structured way. Because it does not rely on absolute perfusion thresholds, it may be less susceptible to software-dependent variability. Whether this facilitates its use across centres remains to be demonstrated, since the score was developed and tested at a single centre with a single scanner and post-processing platform. The interrater agreement for the detection of a perfusion deficit was excellent in our cohort (κ = 0.93, 95% CI 0.90–0.97), whereas the reproducibility of the numerical score itself has not yet been established.

The temporal distribution of perfusion abnormalities observed in our study is consistent with prior work showing that perfusion disturbances typically accumulate between days 4 and 10 after aSAH [27,28]. In our cohort, the perfusion burden reached its maximum around day eight. This slightly later peak may result from delayed imaging in sedated or comatose patients, in whom CT perfusion is often initiated based on increased transcranial Doppler velocities instead of clinical deterioration.

Our findings align with studies showing that extensive perfusion abnormalities are associated with DCI and delayed infarction [10,20,27]. The dichotomous grading of perfusion deficits was strongly associated with infarction but not with functional outcome, potentially reflecting the additional impact of infarct volume and location. This interpretation is supported by recent endovascular series, in which treatment decisions for severe vasospasm were guided by the extent of haemodynamic compromise and downstream perfusion impairment rather than by angiographic narrowing alone [29].

In contrast, the territorial perfusion burden remained independently associated with functional outcome after adjustment for age and clinical grade, suggesting that the extent and regional distribution of hypoperfusion carry prognostic information that a binary assessment does not capture. This comparison of two separate models describes a difference in the pattern of association. It does not formally demonstrate incremental prognostic value, which would require a comparison of model discrimination and calibration.

Similar observations have been reported for territorially extensive or multifocal perfusion impairment, which correlates with larger infarctions and poorer outcomes [15,24,30], and a recent study found that in patients with early perfusion deficits, subsequent infarction developed within the predicted vascular territory in 85% of cases [31].

Treatment modality differed between patients with and without perfusion deficits, with more endovascular treatment and less clipping in the deficit group. This is most likely explained by aneurysm location, as middle cerebral artery aneurysms, which are more often clipped, were less frequent among patients with perfusion deficits, whereas internal carotid artery aneurysms were more frequent. Treatment modality was part of the prespecified covariate set and was not associated with either endpoint (Table 2 and Table 3); nevertheless, residual confounding by treatment-related factors cannot be excluded.

Several studies have emphasized the value of CTP in guiding treatment decisions during the vasospasm period. Sanelli et al. demonstrated that positive perfusion findings substantially increase the post-test probability of DCI and may support the initiation of induced hypertension or intra-arterial therapy, whereas negative findings justify conservative management [32,33]. Heitkamp et al. showed that adding CTP to CTA improves interrater reliability and results in more consistent decisions regarding endovascular therapy [34], and in current rescue-therapy protocols, territorial or watershed perfusion deficits on CTP are used to diagnose DCI in patients who cannot be assessed clinically [16]. In this context, the TPBS may offer an additional structured measure for identifying patients with a high ischaemic burden who may benefit from intensified monitoring or earlier treatment escalation, particularly when clinical examination is limited. Under the assumption of a linear relationship on the logit scale, a maximum score of 6 corresponds to approximately 2.6-fold higher odds of poor functional outcome and 6.3-fold higher odds of new infarction compared with a score of 0. However, treatment decisions remain multifactorial, and no validated threshold for the burden of perfusion impairment currently exists. We deliberately refrained from deriving a cut-off from our data, because a threshold from a single-centre retrospective cohort would be prone to overfitting and would require external validation before it could inform treatment decisions. The score should therefore be regarded as a tool for risk stratification rather than as a standalone determinant for intervention.

The observed associations cannot be interpreted independently of the treatment that followed the imaging findings. Patients with perfusion deficits underwent intra-arterial spasmolysis substantially more often (73.8% vs. 28.1%), because the perfusion finding itself was one of the triggers for treatment escalation. Treatment may in turn have altered the perfusion trajectory and the probability of infarction and poor outcome. We did not adjust for spasmolysis. It is a consequence of the perfusion finding and a possible cause of the outcome, i.e., not a confounder, and adjusting for it would remove part of the association that the score is meant to capture rather than confounding [18,19]. If spasmolysis was effective, its preferential use in patients with a high perfusion burden would attenuate rather than inflate the reported associations. The reported odds ratios therefore describe the prognostic association of the perfusion burden under the treatment strategy applied at our institution and not the natural course of untreated hypoperfusion.

This study has several limitations. First, it is a retrospective single-centre analysis with the inherent risks of missing data and selection bias. CTP was not obtained in every patient but according to clinical indication, i.e., in patients with neurological deterioration, increased TCD velocities, or an inability to be assessed clinically because of sedation or intubation. Patients with an uncomplicated course were therefore underrepresented, and the prevalence of perfusion deficits (68%) and of new infarction (51.2%) must not be interpreted as representative of an unselected aSAH population. This does, however, reflect the clinical reality in many hospitals. Second, the maximum TPBS depends on the number and timing of examinations. Patients who were examined more often had more opportunity to reach a high score, and imaging frequency was itself related to the severity of the clinical course, so that surveillance intensity and disease severity cannot be separated in this design. The binary deficit variable is subject to the same mechanism. Third, for infarction at discharge we cannot exclude that in some patients the maximum score was reached after the infarction had already developed. This association should therefore be read as a radiological correlate rather than as a prediction, whereas the temporal sequence for functional outcome at 6 months is unambiguous. Fourth, the multivariable models were built by forward selection from a prespecified set of candidate variables, which can yield optimistic estimates, although the TPBS estimate changed little between univariable and multivariable analysis. A formal comparison of model discrimination was not performed, and the score was entered as a linear term, so that a nonlinear relationship at the upper end of the score cannot be excluded. Fifth, interrater agreement was assessed for the presence of a perfusion deficit and not for the numerical score, and the visual definition of delayed perfusion retains a subjective component. Last, patients with perfusion deficits underwent intra-arterial spasmolysis substantially more often, reflecting treatment escalation in response to imaging findings. This introduces treatment-by-indication bias and may have influenced both perfusion dynamics and clinical outcome, which cannot be fully accounted for in a retrospective study design.

In view of these limitations, our findings should be regarded as preliminary and hypothesis generating. They indicate that the extent of perfusion impairment carries prognostic information in patients under CTP surveillance, but they do not establish the score as a validated prognostic tool. Prospective studies with predefined imaging time points, assessment of clinical DCI according to consensus criteria, evaluation of the interrater reliability of the numerical score, a lesion-level comparison of infarct location with the previously affected territories, and a comparison of model discrimination against clinical and binary perfusion models are required before the TPBS can be recommended for clinical decision-making. External validation in other centres is a prerequisite for any claim of multicentre applicability.

## 5. Conclusions

In this retrospective single-centre cohort of patients with aSAH under indication-driven CTP surveillance, the territorial perfusion burden score was independently associated with new cerebral infarction and with poor functional outcome, whereas the binary classification of a perfusion deficit was associated with infarction only. These preliminary findings suggest that the extent, and not only the presence, of perfusion impairment is relevant for risk stratification during the vasospasm period. Prospective validation with standardized imaging time points and in external cohorts is required before the score can be used to guide clinical decisions.

## Figures and Tables

**Table 1:** Baseline characteristics, aneurysm features, treatment details, and clinical outcomes of patients with aneurysmal subarachnoid haemorrhage stratified by the presence of computed tomography perfusion deficits (*n* = 179).

Characteristic	Total (*n* = 179)	Perfusion Deficits (*n* = 122)	No Deficits (*n* = 57)	*p*-Value
Baseline characteristics				
Age, years	57.5 (13.0)	56.8 (13.3)	59.0 (12.3)	0.28
Female sex	68 (38%)	44 (36.1%)	24 (42.1%)	0.51
Diabetes mellitus	7 (3.9%)	5 (4.1%)	2 (3.5%)	1.00
Hypertension	84 (46.9%)	60 (49.2%)	24 (42.1%)	0.42
Current smoker	66 (36.9%)	46 (37.7%)	20 (35.1%)	0.87
Ruptured aneurysm characteristics				
Aneurysm location				
Anterior circulation	145 (81.0%)	101 (83.6%)	45 (78.9%)	0.54
ICA	19 (10.6%)	17 (13.9%)	2 (3.5%)	0.04 ^†^
ACA	75 (41.9%)	54 (44.3%)	20 (35.1%)	0.25
MCA	30 (16.8%)	13 (10.7%)	15 (26.3%)	0.01 ^†^
PCom	26 (14.5%)	17 (13.9%)	8 (14.0%)	0.99
Posterior circulation	34 (19.0%)	21 (16.4%)	12 (21.1%)	0.54
Aneurysm size, mm	6.2 (3.4)	6.5 (3.4)	5.6 (3.4)	0.09
Haemorrhage severity				
mFisher	3 (2–4)	3 (2–4)	3 (2–4)	0.84
ICH at admission	61 (34.2%)	44 (36.1%)	17 (29.8%)	0.50
Clinical severity				
Hunt and Hess	3 (2–4)	3 (2–4)	3 (1–4)	0.68
Treatment				
Endovascular treatment	128 (71.5%)	97 (79.5%)	31 (54.4%)	<0.001 ^†^
Surgical clipping	52 (28.5%)	25 (20.5%)	26 (45.6%)	<0.001 ^†^
EVD	140 (78.2%)	95 (77.9%)	45 (78.9%)	1.00
Intra-arterial spasmolysis	106 (59.2%)	90 (73.8%)	16 (28.1%)	<0.001 ^†^
Clinical outcome				
New infarction at discharge	88 (51.2%)	71 (61.2%)	17 (30.4%)	<0.001 ^†^
Good outcome (mRS ≤ 2)	79 (45.9%)	59 (50.0%)	20 (37.0%)	0.14
In-hospital mortality	23 (12.8%)	17 (13.9%)	6 (10.5%)	0.64

Continuous variables are presented as mean ± standard deviation for normally distributed data or median with interquartile range for non-normally distributed data. Categorical variables are presented as number (percentage). Group comparisons were performed using the χ^2^ test for categorical variables, the unpaired *t*-test for continuous variables, and the median test for ordinal variables. ICA = internal carotid artery; ACA = anterior cerebral artery; MCA = middle cerebral artery; PCom = posterior communicating artery; mFisher = modified Fisher grade; ICH = intracerebral haemorrhage; EVD = external ventricular drainage; mRS = modified Rankin Scale. ^†^ *p* < 0.05. Percentages for functional outcome and new infarction were calculated based on patients with available outcome data.

**Table 2:** Logistic regression analysis of poor functional outcome at 6-month follow-up (mRS ≥ 3; *n* = 172).

Variable	Univariable OR (95% CI)	Adjusted OR, TPBS Model	Adjusted OR, Deficit Model
Age	1.05 (1.02–1.07)	1.05 (1.02–1.08)	1.04 (1.01–1.07)
Hunt and Hess	1.98 (1.53–2.57)	1.90 (1.45–2.48)	1.73 (1.29–2.30)
mFisher	2.04 (1.48–2.81)	NR	1.51 (1.04–2.18)
ICH at admission	2.84 (1.48–5.45)	NR	NR
Surgical clipping	1.12 (0.58–2.18)	NR	NR
Necessity of EVD	5.14 (2.12–12.50)	NR	NR
Dichotomized perfusion deficit	1.70 (0.88–3.29)	-	NR
TPBS	1.18 (1.05–1.32)	1.17 (1.02–1.33)	-

Odds ratios are presented with 95% confidence intervals; odds ratios for the TPBS are given per one-point increase. Multivariable models were built by forward conditional selection from the prespecified candidate covariates (age, Hunt and Hess grade, modified Fisher grade, intracerebral haemorrhage at admission, treatment modality, and external ventricular drainage). The binary perfusion deficit and the territorial perfusion burden score were analysed in separate models due to collinearity. TPBS = territorial perfusion burden score; EVD = external ventricular drainage; ICH = intracerebral haemorrhage; mFisher = modified Fisher grade; mRS = modified Rankin Scale; NR = not retained in the final model.

**Table 3:** Logistic regression analysis of new cerebral infarction at discharge (*n* = 172).

Variable	Univariable OR (95% CI)	Adjusted OR, TPBS Model	Adjusted OR, Deficit Model
Age	1.01 (0.99–1.04)	NR	NR
Hunt and Hess	1.10 (0.87–1.37)	NR	NR
mFisher	1.19 (0.89–1.60)	NR	NR
ICH at admission	1.09 (0.56–2.11)	NR	NR
Surgical clipping	0.57 (0.27–1.20)	NR	NR
Necessity of EVD	1.30 (0.59–2.87)	NR	NR
Dichotomized perfusion deficit	8.88 (3.31–23.87)	-	8.88 (3.31–23.87)
TPBS	1.36 (1.19–1.55)	1.36 (1.19–1.55)	-

Odds ratios are presented with 95% confidence intervals; odds ratios for the TPBS are given per one-point increase. Multivariable models were built by forward conditional selection from the prespecified candidate covariates (age, Hunt and Hess grade, modified Fisher grade, intracerebral haemorrhage at admission, treatment modality, and external ventricular drainage). The binary perfusion deficit and the territorial perfusion burden score were analysed in separate models due to collinearity. TPBS = territorial perfusion burden score; EVD = external ventricular drainage; ICH = intracerebral haemorrhage; mFisher = modified Fisher grade; NR = not retained in the final model.

## Data Availability

The data presented in this study are available on request from the corresponding author. The data are not publicly available due to privacy restrictions.

## Abbreviations

The following abbreviations are used in this manuscript:

aSAH	aneurysmal subarachnoid haemorrhage
DCI	delayed cerebral ischaemia
CTA	computed tomography angiography
CTP	computed tomography perfusion
TPBS	territorial perfusion burden score
mRS	modified Rankin Scale
EVD	external ventricular drainage
ICH	intracerebral haemorrhage
TCD	transcranial Doppler
DSA	digital subtraction angiography
CBF	cerebral blood flow
CBV	cerebral blood volume
MTT	mean transit time
TTD	time-to-drain
OR	odds ratio
CI	confidence interval
IQR	interquartile range
SD	standard deviation
ACA	anterior cerebral artery
MCA	middle cerebral artery
PCA	posterior cerebral artery
ICA	internal carotid artery
PCom	posterior communicating artery
